# Clinical Efficacy and Psychological Mechanisms of an App-Based Digital Therapeutic for Generalized Anxiety Disorder: Randomized Controlled Trial

**DOI:** 10.2196/26987

**Published:** 2021-12-02

**Authors:** Alexandra Roy, Elizabeth A Hoge, Pablo Abrante, Susan Druker, Tao Liu, Judson A Brewer

**Affiliations:** 1 Department of Behavioral and Social Sciences Brown University School of Public Health Providence, RI United States; 2 Department of Psychiatry Georgetown University Medical Center Washington, DC United States; 3 Department of Population and Quantitative Health Sciences University of Massachusetts Medical School Worcester, MA United States; 4 Department of Biostatistics Brown University School of Public Health Providence, RI United States

**Keywords:** anxiety, generalized anxiety disorder, worry, mindfulness, mHealth, digital therapeutics, mobile phone

## Abstract

**Background:**

Current treatments for generalized anxiety disorder (GAD) often yield suboptimal outcomes, partly because of insufficient targeting of underlying psychological mechanisms (eg, avoidance reinforcement learning). Mindfulness training (MT) has shown efficacy for anxiety; yet, widespread adoption has been limited, partly because of the difficulty in scaling in-person–based delivery. Digital therapeutics are emerging as potentially viable treatments; however, very few have been empirically validated.

**Objective:**

The aim of this study is to test the efficacy and mechanism of an app-delivered MT that was designed to target a potential mechanism of anxiety (reinforcement learning), based on which previous studies have shown concern regarding feedback and the perpetuation of anxiety through negative reinforcement.

**Methods:**

Individuals with GAD were recruited using social media advertisements and randomized during an in-person visit to receive treatment as usual (n=33) or treatment as usual+app−delivered MT (Unwinding Anxiety; n=32). The latter was composed of 30 modules to be completed over a 2-month period. Associated changes in outcomes were assessed using self-report questionnaires 1 and 2 months after treatment initiation.

**Results:**

We randomized 65 participants in this study, and a modified intent-to-treat approach was used for analysis. The median number of modules completed by the MT group was 25.5 (IQR 17) out of 30; 46% (13/28) of the participants completed the program. In addition, the MT group demonstrated a significant reduction in anxiety (GAD-7) compared with the control group at 2 months (67% vs 14%; median change in GAD-7: –8.5 [IQR 6.5] vs –1.0 [IQR 5.0]; *P*<.001; 95% CI 6-10). Increases in mindfulness at 1 month (nonreactivity subscale) mediated decreases in worry at 2 months (Penn State Worry Questionnaire; *P*=.02) and decreases in worry at 1 month mediated reductions in anxiety at 2 months (*P*=.03).

**Conclusions:**

To our knowledge, this is the first report on the efficacy and mechanism of an app-delivered MT for GAD. These findings demonstrate the clinical efficacy of MT as a digital therapeutic for individuals with anxiety (number needed to treat=1.6). These results also link recent advances in our mechanistic understanding of anxiety with treatment development, showing that app-delivered MT targets key reinforcement learning pathways, resulting in tangible, clinically meaningful reductions in worry and anxiety. Evidence-based, mechanistically targeted digital therapeutics have the potential to improve health at a population level at a low cost.

**Trial Registration:**

ClinicalTrials.gov NCT03683472; https://clinicaltrials.gov/ct2/show/NCT03683472

## Introduction

### Background

Anxiety disorders are the most common class of mental illnesses, with a 31% lifetime prevalence [1]. This has already increased during the COVID-19 pandemic [2-4]. For example, in the United States, the Census Bureau reported that adults were more than 3 times more likely to screen positive for an anxiety disorder in 2020 than they were in 2019 (31% vs 8%), and a cross-sectional survey of people in China in 2020 reported the prevalence of generalized anxiety disorder (GAD) to be 35.1% [2]. A recent meta-analysis of 17 studies (N=62,000) found an average prevalence of anxiety of 32% during COVID-19 [5].

### Treatment for Anxiety

However, anxiety, particularly GAD, is difficult to treat. Current practice guidelines recommend pharmacological and psychological interventions [6], but most patients favor psychotherapy over medications [7]. Benzodiazepines have the risk of tolerance and addiction; the United Kingdom National Institute for Health and Care Excellence guidelines state that benzodiazepines “should not be used routinely to treat anxiety disorders” [6]. First-line treatment for anxiety, such as selective serotonin reuptake inhibitors, can have limitations, including delayed patient responses and adverse effects (eg, gastrointestinal and sexual) [8,9]. The number needed to treat (NNT) for selective serotonin reuptake inhibitors is 5.2; one needs to treat >5 individuals to see a significant response in 1 individual [10].

Cognitive behavioral therapy (CBT), the most commonly used and researched psychological intervention for anxiety, has been associated with reduction in symptoms using measures such as the Beck Anxiety Inventory (small to medium effect sizes), and yet is typically delivered in-person [11]. Given the growing need and shortage of trained therapists [12,13], new mechanistically based treatments that can be delivered at scale and a distance are needed for those unable to attend in person because of barriers such as living in a resource-limited area or unwillingness to go to a mental health clinic because of stigma. In addition, treatments that target more recent mechanistic underpinnings of anxiety are needed.

### Psychological Mechanisms of Anxiety

From a theoretical standpoint, reinforcement learning mechanisms have been suggested to drive anxiety disorders [14,15]. Worry is widely regarded as the central defining feature of GAD and has been shown to be triggered as an avoidance reaction to emotional experiences [16], thus learned as “a negatively reinforced avoidant behavior” [17]. Recent research has linked reinforcement learning with biological mechanisms and clinical symptoms [18]. Worry represents an attempt to engage in mental problem solving on an issue with an uncertain outcome [19]. Although worry is unpleasant, the immediate emotions that are avoided by focusing on worry, such as fear, are often perceived as *more* unpleasant [20,21]. Therefore, aversive stimuli can trigger worry as an avoidant behavior, which then becomes habitual [22]. Theoretically and mechanistically, worry is learned and reinforced in a manner similar to other types of operantly conditioned behaviors [14]. With cycles of reinforcement learning, when triggered by feelings of anxiety, individuals with GAD learn a maladaptive thinking style that uses worry as a focus on the future rather than the present. As described by Borkovec et al [14,15], when worry becomes habitual, its negative reinforcement pathway can spiral out of control; when the negative emotional experience of worry rises to the same level as unpleasant emotional states that trigger it, it may become its own trigger for more anxiety, which leads to more worry directly driving anxiety *habit loops* (Gao et al, unpublished data, 2021).

In conventional frameworks, anxiety is conceptualized as an overestimation of danger and an underestimation of one’s ability to cope with it [23]. Cognitive therapies aim to interrupt the cycle of worry by replacing maladaptive cognitions with more constructive ones. For individuals who cycle into anxious worry to a degree that impairs prefrontal cortical function and the ability to use cognitive therapies [24], other strategies are needed. New treatments such as mindfulness training (MT) have shown promise in efficacy and cost, with effect sizes rivaling current treatments [25]. Furthermore, cross-sectional studies of nonclinical populations have suggested a mediating role of worry in the effects of MT on anxiety [26].

### Mindfulness

Mindfulness can be defined as the awareness that arises when paying attention to the present moment on purpose and nonjudgmentally [27]. The attitudinal quality of not judging and allowing experience to unfold with curiosity targets maladaptive reinforcement learning by helping individuals to simply observe repetitive cycles of perseverative worry rather than to habitually react and reinforce them [28]. MT has been found to mechanistically break key links in the reinforcement pathway for other habitual behaviors such as smoking and emotional eating [29,30], with concomitant changes in related brain regions predicting clinical outcomes [31]. Worry has been shown to activate brain networks associated with self-referential processing, such as the default-mode network, suggesting that the more one is *caught up* in perseveration about uncertain events, the more this network is activated [32,33]; meditation has been shown to directly deactivate these brain regions [34], and neurophenomenological studies suggest that this may be because of the ability to observe thoughts and emotions rather than being caught up in them [35-37]. Specific to GAD, MT has been associated with changes in the fronto-limbic brain regions involved in emotion regulation with simultaneous improvements in reported symptoms [38].

### App-Delivered MT

Regarding treatment delivery, mindfulness-based interventions, such as mindfulness-based stress reduction, are generally delivered in a group format. However, concerns remain regarding the scalability of in-person–delivered treatment [39]. Digital therapeutics (ie, app-delivered interventions) have garnered much attention as a new modality that can deliver high-fidelity treatment at scale and low cost. However, empirically tested apps are not widely used, and widely used apps do not have an evidence base (<0.05%)—a clear “digital research practice gap” [40]. To date, only 1 study has reported the clinical efficacy of a digital therapeutic specifically for people diagnosed with GAD (showing that CBT may be effective when delivered in this format) [41].

To address the digital research practice gap, we designed an app-delivered digital therapeutic program for anxiety that mechanistically targets reinforcement learning using MT to help individuals identify habitual worry thinking patterns and learn not to habitually react to unpleasant emotions (ie, break the worry cycle). In a single-arm study of anxious physicians, we found preliminary evidence for its utility in reducing anxiety (57% reduction in GAD-7 scores after 3 months) [42]. However, randomized controlled studies are required to determine the efficacy and mechanisms of action. We tested the following hypotheses in a randomized controlled trial of individuals with GAD: (1) app-delivered MT would show superior efficacy in reducing anxiety and worry than standard treatment; (2) increases in nonreactivity would mediate reductions in worry; and (3) reductions in worry would mediate reductions in anxiety.

## Methods

### Study Overview and Participants

We used a parallel-group randomized controlled trial design with analyses and outcome measures preregistered at ClinicalTrials.gov. Individuals were recruited using Facebook advertisements and screened for eligibility via a phone call by the project director (AR). Textbox 1 details the inclusion and exclusion criteria. These criteria were chosen to mimic, as closely as possible, real-world clinical situations while minimizing potential confounders (eg, a recent change in medication dose may mask treatment effects). Eligible participants attended an in-person research visit at Brown University, where they underwent informed consent procedures with the project director before enrolling in the study. Participants were provided Amazon gift cards worth up to US $80 to complete the questionnaires. This study was approved by the Institutional Review Board of Brown University.

**Inclusion and exclusion criteria.**

**Inclusion criteria**

- Score ≥10 on the Generalized Anxiety Disorder (GAD) 7-item scale, which is suggestive of a diagnosis of GAD (sensitivity and specificity of 89% and 82%, respectively) [43]
- Owns a smartphone
- Willingness to receive check-in calls
- 18 years or older

**Exclusion criteria**

- Dose changes for any psychoactive medication in the last 2 months
- As-needed use of benzodiazepines or hypnotic sleep aids
- History of bipolar, schizophrenia, schizoaffective, or another psychotic disorder
- Significant medical condition that would impact the ability to complete study tasks
- Cohabitation with someone already enrolled in the study
- Previous use of other related apps

After enrolling participants in the study, the project director, who had previously undergone training and was supervised by a psychiatrist, conducted an in-person, abbreviated version of the Mini-International Neuropsychiatric Interview to confirm a diagnosis of GAD, along with the assessment of other potential comorbid disorders, such as major depressive episode, panic disorder, agoraphobia, social anxiety disorder, obsessive compulsive disorder, and posttraumatic stress disorder [44]. Participants were then asked to complete a web-based questionnaire via Qualtrics [45], which included demographic and self-report items. Participants in both groups received questionnaires via email 1 and 2 months after treatment initiation. Upon completion of the final questionnaire, the treatment as usual (TAU) group received instructions on how to download and install the app.

### Randomization and Blinding

After completing the baseline questionnaire, participants were given a sealed, opaque envelope (prepared, reviewed, and sealed by individuals independent of the study team) by the project director that contained their group assignment: TAU+app-delivered MT or TAU. The 1:1 randomization scheme was generated by an independent statistician with variable block sizes of 4 and 6. Team members who randomized the participants and carried out the study procedures did not perform the study analyses. The principal investigator and the statistician who conducted the statistical analysis were blinded to the group allocation until all analyses were complete.

### Intervention

The app-delivered MT program, Unwinding Anxiety (version 2), is a Health Insurance Portability and Accountability Act compliant digital therapeutic that teaches individuals to understand how anxious worry develops and perpetuates through reinforcement learning and how to bring mindful awareness to moments of stress and worry so that they can observe feelings of anxiety rather than perpetuate reactive worry thinking. This process helps individuals *unlearn* or extinguish worry at the core mechanistic level. This experiential education is delivered via a smartphone-based platform, which includes a progression through >30 daily modules of brief didactic and experience-based MT (videos and animations approximately 10 minutes per day; Multimedia Appendix 1), app-triggered check-ins, user-initiated guided meditations (5-15 minutes), and brief (30 seconds) on-demand mindfulness exercises to help disrupt anxiety cycles in vivo (Textbox 2; Multimedia Appendix 2). The content for this intervention was developed based on a combination of clinical experiences and previously developed, in-person and app-delivered MT protocols for habit change that have yielded clinically meaningful outcomes (eg, smoking and overeating) [28-30,46-49]; an open-label pilot study of the app demonstrated a 57% reduction in GAD-7 scores in anxious physicians [42].

**Overview of Unwinding Anxiety themes and content.**

**Modules 1-7 (goals, curiosity, reinforcement learning, body scan, and self-monitoring)**

- Set goals and introduce how habits are formed around worry (eg, reinforcement learning and distraction); introduce curiosity to foster the nonjudgmental aspects of mindfulness and basic mindfulness practices, including the body scan; and unpack worry and fear both from a brain and behavior perspective.

**Modules 8-14 (noting practice; RAIN [recognize, accept, investigate, and note]; barriers to change; and reinforcement of concepts)**

- Introduce how to mindfully work with worry cues and affective states using RAIN (recognize, accept, investigate, and note what emotions feel like as they arise and pass away); build on basic mindfulness using noting practice (the N of RAIN) during everyday life; and introduce additional animations to reinforce mindfulness concepts that show how we feed our anxiety by worry thinking and distraction.

**Modules 15-21 (noting practice [continued from previous modules]; RAIN [continued from previous modules]; thinking versus knowing; and *un*resistance)**

- Reinforce noting practice and continue to train and support self-kindness; specifically address the difference between trying to think our way out of uncertainty (or anxiety) and resting in a kind, curious awareness of it; and focus on not resisting experience and not getting tripped up by worry thinking.

**Modules 22-30 (noting practice [continued from previous modules]; RAIN [continued from previous modules]; and working with uncertainty and change)**

- Help individuals reflect on their own evidence base for working with worry to solidify their shift from reactivity to mindfully being with emotions as a new habit.

**Modules 30 and onward (reinforcing concepts via *theme weeks* and individual customization via *personal week*)**

- >8 themed weeks and unlimited personalization of content by picking modules to develop a custom week for review.

### Intervention Orientation and Engagement

Individuals randomized to TAU+app-delivered MT were assisted with the installation of the app on their smartphone and the reviewing of the features. They were instructed to complete 1 module per day over the subsequent 30 days at a time of their choice. In addition, the intervention would check in with them 3 times throughout the day (this could be modified by the user) and offer brief mindfulness exercises. Participants were encouraged to use other app features but were informed that this was not a requirement for the study. The project director sent check-in messages on days 3, 7, 14, and 21 from treatment initiation to help mitigate technical difficulties and encourage engagement. Specifically, participants were asked “how things were going with the app since the last time they received a check-in.” If the participant expressed difficulties, efforts were made to resolve the problem.

### TAU Condition

As part of TAU, participants were asked to continue the standard care set forward by their clinician or clinicians. This could include pharmacological treatment or psychotherapy. Participants were also provided with a list of local resources.

### Outcome Measures

The primary outcomes were changes in anxiety, as measured by the GAD-7, and emotional reactivity at 2 months after treatment initiation. Secondary outcomes included changes in worry, as measured by the Penn State Worry Questionnaire (PSWQ) and interoceptive awareness.

#### GAD 7-Item

GAD-7 is a validated 7-item questionnaire used clinically to screen for probable diagnosis of GAD (sensitivity of 89% and specificity of 82%; high internal consistency, with Cronbach α=.92) and track symptom severity [43]. Individuals are asked, “in the last week, how often have you been bothered by the following problems” with prompts such as *feeling nervous, anxious, or on edge* and *trouble relaxing* [50]. The scale ranges from 0 (*not at all sure*) to 3 (*nearly every day*), with scores ranging from 0 to 21 [50]. Total scores of 5, 10, and 15 serve as cut-off points for mild, moderate, and severe anxiety, respectively; therefore, remission is a score of ≤4 [50]. The minimal clinically important difference for GAD-7 was 3.8; the clinically relevant change was ≥4 points [51]. GAD-7, which is highly correlated with the Hamilton Anxiety Scale (*r*=0.852), was used based on its *real-world* utility, as it is the most commonly employed tool in primary care and other outpatient settings and has been incorporated into most large-scale electronic medical record systems [52].

#### Five Facet Mindfulness Questionnaire Nonreactivity Subscale

The nonreactivity subscale is composed of 7 questions from the 39-item Five Facet Mindfulness Questionnaire (FFMQ) with acceptable internal consistency (Cronbach α=.75) [53]. It is validated for use independently and assesses nonreactivity to inner experience [54]. Individuals are asked questions about what is generally true for them on a scale from 1 (*never or very rarely true*) to 5 (*very often or always true*) [53]. Examples include “I perceive my feelings and emotions without having to react to them” and “When I have distressing thoughts, I feel calm soon after” [53]. Scores range from 7 to 35, with higher scores indicating an increase in nonreactivity.

#### Penn State Worry Questionnaire

The PSWQ is a validated 16-item questionnaire with high internal consistency (Cronbach α=.93) used to assess worry [55]. Individuals are asked to rate statements on a scale ranging from 1 (*not at all typical of me*) to 5 (*very typical of me*) [55]. Example items include “My worries overwhelm me” and “When I am under pressure I worry a lot” [55]. Scores range from 16 to 80, with higher scores indicating a higher degree of worry.

#### Multidimensional Assessment of Interoceptive Awareness

The Multidimensional Assessment of Interoceptive Awareness (MAIA) is a 32-item questionnaire that assesses 8 domains of interoceptive awareness: noticing, not distracting, not worrying, attention regulation, emotional awareness, self-regulation, body listening, and trusting (Cronbach α=.66-.87) [56]. On a scale of 0 (*never*) to 5 (*always*), individuals are asked how often each statement applies to them generally in daily life [56]. Statements include “I distract myself from sensations of discomfort” and “I trust my body sensations” [56]. Scores range from 0 to 160, with higher scores indicating greater interoceptive awareness.

#### Safety and Adverse Events

Monitoring of safety occurred continuously during the study, and if an adverse event was reported, follow-up was conducted via phone by the project director using National Institute of Mental Health reportable events templates [57]. A final check was conducted by the project director at the conclusion of the study to assess adverse events potentially related to the intervention. If an event was reported, the same process was followed.

### Sample Size

As there are no prior studies using the intervention, the target sample size of 65 was determined using pilot data. A pilot study of individuals with GAD-7 scores >9 (n=17) demonstrated a 39% reduction in the scores after completing 21 modules. Assuming a 10% reduction in the TAU group, a 1-tailed *t* test determined that a sample size of 52 would have 80% power with 1-sided 5% type I error to detect a statistically significant between-group difference in GAD-7 scores (Cohen *d*=0.7). We recruited 65 individuals to account for 25% attrition.

### Statistical Analysis

Analyses were conducted using R (version 3.4.1), and a modified intent-to-treat approach was employed. This was defined as all participants who downloaded the app and completed all study assessments, regardless of treatment completion. A robust, mixed analysis of variance (ANOVA) was used to evaluate the primary outcomes, changes in anxiety, and emotional reactivity 2 months after treatment initiation in the TAU+app-delivered MT group relative to those in the TAU group. This and other robust statistical tests were chosen to avoid violating underlying model assumptions, such as normality. Because of the use of these nonparametric tests, the median and IQR were calculated. Post hoc comparisons of single effects were performed using the WRS2 package in R. In addition, Mann-Whitney *U* tests were performed, and the Hodges-Lehmann estimate of location shift was used to calculate the difference between groups at each time point [58]. Bonferroni correction was used to adjust for multiple comparisons. Effect sizes (*r*) were calculated by dividing the *z* score by the square root of the sample size using the Cohen criteria for *r,* where 0.1 is small, 0.3 is medium, and 0.5 is large [59].

**Figure 1:**
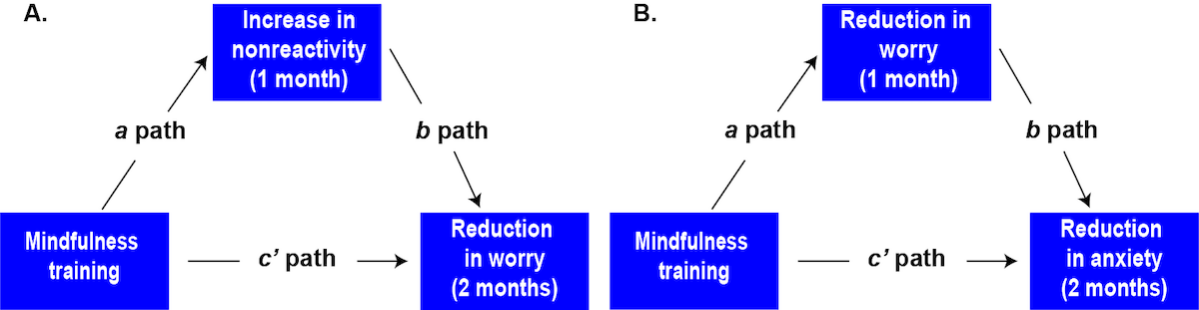
(A) Path model for longitudinal causal mediation evaluating if increases in nonreactivity mediate the relationship between MT and reduction in worry. (B) Path model for longitudinal causal mediation evaluating if reduction in worry mediates the relationship between MT and reduction in anxiety. MT: mindfulness training.

We then conducted exploratory mediation analyses to evaluate (1) model A, whether increased nonreactivity mediated the relationship between MT and reduced worry, and (2) model B, whether reduced worry mediated the relationship between MT and reduced anxiety. To reduce the impact of the unit on the comparison of direct and indirect effects, the variables were standardized to have 0 means and unit SD. To have causal interpretations, the mediation models were built on the longitudinal [60]: In model A, MT was the independent variable, worry was the dependent variable, and nonreactivity was the mediating variable (Figure 1); in model B, MT was the independent variable, anxiety was dependent variable, and worry was the mediating variable (Figure 1). We calculated the direct and indirect effects, for which the SEs and 95% CI were computed using the bootstrap method with 1000 bootstrapped resamples [61].

The median and IQR were calculated to evaluate the engagement or the total number of modules completed. To explore the impact of anxiety on engagement at 2 months after treatment initiation, a robust regression model based on an M-estimator, which uses iteratively reweighted least squares estimation, was fitted, with the anxiety score as the independent variable and the total number of modules completed as the dependent variable.

### Number Needed to Treat

The NNT, defined as the total number of individuals who need to receive treatment to prevent 1 adverse event, is a standard epidemiological measure used to communicate the effectiveness of a health care intervention [62]. The inverse of the absolute risk reduction was calculated by subtracting the total percentage of individuals who achieved remission (GAD-7 score ≤5, minimal anxiety) in the TAU group from the total percentage of those in the TAU+app-delivered MT group.

### Reliable Change Index

Unlike statistical significance, clinical significance has traditionally lacked a consistent definition [63]. To address this need, a reliable change index (RCI) was created to evaluate the reliability of clinically significant changes [63]. We used the method developed by Jacobson and Truax to calculate the RCI for changes in anxiety scores [64]. If the RCI exceeded the *z*-scored level of significance from –1.96 to +1.96 (*P*<.05), we evaluated the percentage of participants with clinically significant change who met or exceeded it at 1 and 2 months after treatment initiation [63].

### Risk of Bias

Six areas of potential bias across 7 domains were assessed using the Cochrane Collaboration tool for evaluating the risk of bias [65].

## Results

### Participants

**Figure 2:**
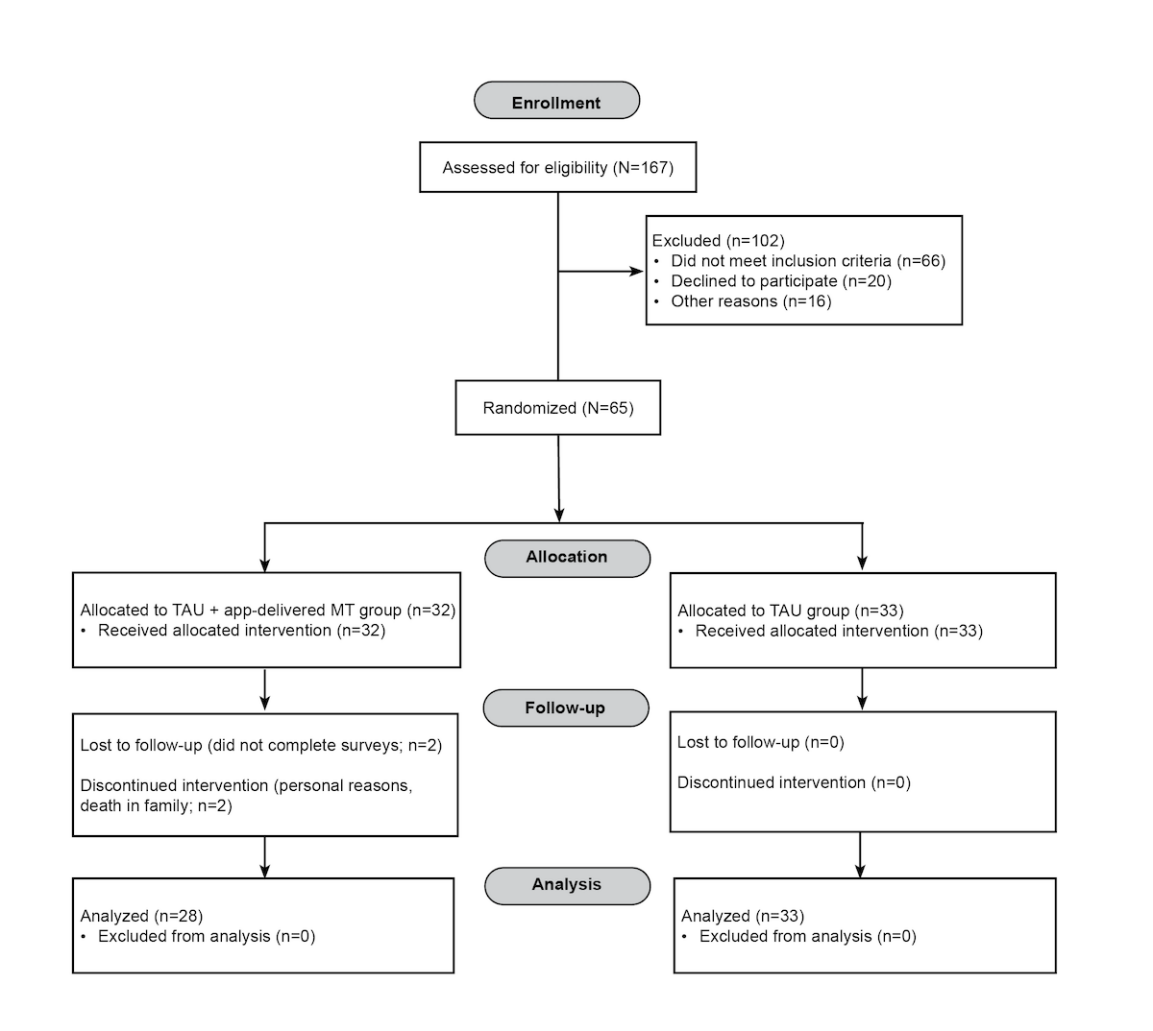
Participant flow diagram. MT: mindfulness training; TAU: treatment as usual.

We recruited 65 participants, obtained their consent to participate, and randomized them between May 2019 and October 2019. Baseline demographic characteristics are reported in Table 1. Of the 65 participants, 61 completed the study and were included in the modified intent-to-treat analysis (Figure 2). Before treatment initiation, 30% (19/63), 25% (16/63), and 32% (20/63) of participants reported comorbid anxiety, depression, and anxiety and depressive disorders, respectively (Table 1).

**Table 1:** Baseline demographic characteristics (N=63).

Characteristics		TAU^a^+app-delivered MT^b^ participants (n=30)	TAU participants (n=33)
Age (years), mean (SD)		43 (15)	41 (16)
**Sex, n (%)**			
	Male	2 (7)	3 (9)
	Female	28 (93)	29 (88)
	Other	0 (0)	1 (3)
**Highest level of education completed, n (%)**			
	High school graduate or equivalent (eg, GED^c^)	0 (0)	1 (3)
	Some college or technical school	7 (23)	5 (15)
	Associate degree	2 (7)	3 (9)
	Bachelor’s degree	7 (23)	16 (49)
	Master’s degree	13 (43)	8 (24)
	Doctorate	1 (3)	0 (0)
**Work status, n (%)**			
	Full-time	17 (57)	15 (46)
	Part-time	3 (10)	9 (27)
	Unemployed for <1 month	1 (3)	2 (6)
	Unemployed for >1 month	3 (10)	3 (9)
	Never employed	0 (0)	1 (3)
	Not in labor force	6 (20)	3 (9)
**Marital status, n (%)**			
	Never married	9 (30)	13 (39)
	Married or cohabiting	16 (53)	18 (54)
	Separated or divorced	4 (13)	2 (6)
	Widowed	1 (3)	0 (0)
**Race and ethnicity, n (%)**			
	White	27 (90)	28 (85)
	Black	1 (3)	1 (3)
	Asian	0 (0)	1 (3)
	White, American Indian, or Alaskan native	1 (3)	0 (0)
	White and Black	0 (0)	2 (6)
	Hispanic, White, American Indian, or Alaskan native	1 (3)	1 (3)
**Comorbid conditions, n (%)**			
	Anxiety disorder or disorders	10 (33)	9 (27)
	Depressive disorder or disorders	10 (33)	6 (18)
	Anxiety and depressive disorder or disorders	7 (23)	13 (39)
	None	3 (10)	5 (15)
**Concomitant medications, n (%)**			
	Selective serotonin reuptake inhibitors	6 (20)	3 (9)
	SNRIs^d^	3 (3)	3 (9)
	Other	2 (7)	6 (18)
	>1 medication	2 (7)	5 (15)
	None	17 (57)	17 (52)

^a^TAU: treatment as usual.

^b^MT: mindfulness training.

^c^GED: general educational development.

^d^SNRIs: serotonin and norepinephrine reuptake inhibitors.

### Safety

There were no adverse events in the TAU group and 11% (3/28) of adverse events in the TAU+app-delivered MT group (2/3, 66% *anxiety* and 1/3, 33% *back pain*).

### Changes in Outcome Measures

#### Effects of Intervention on Anxiety (GAD-7)

**Figure 3:**
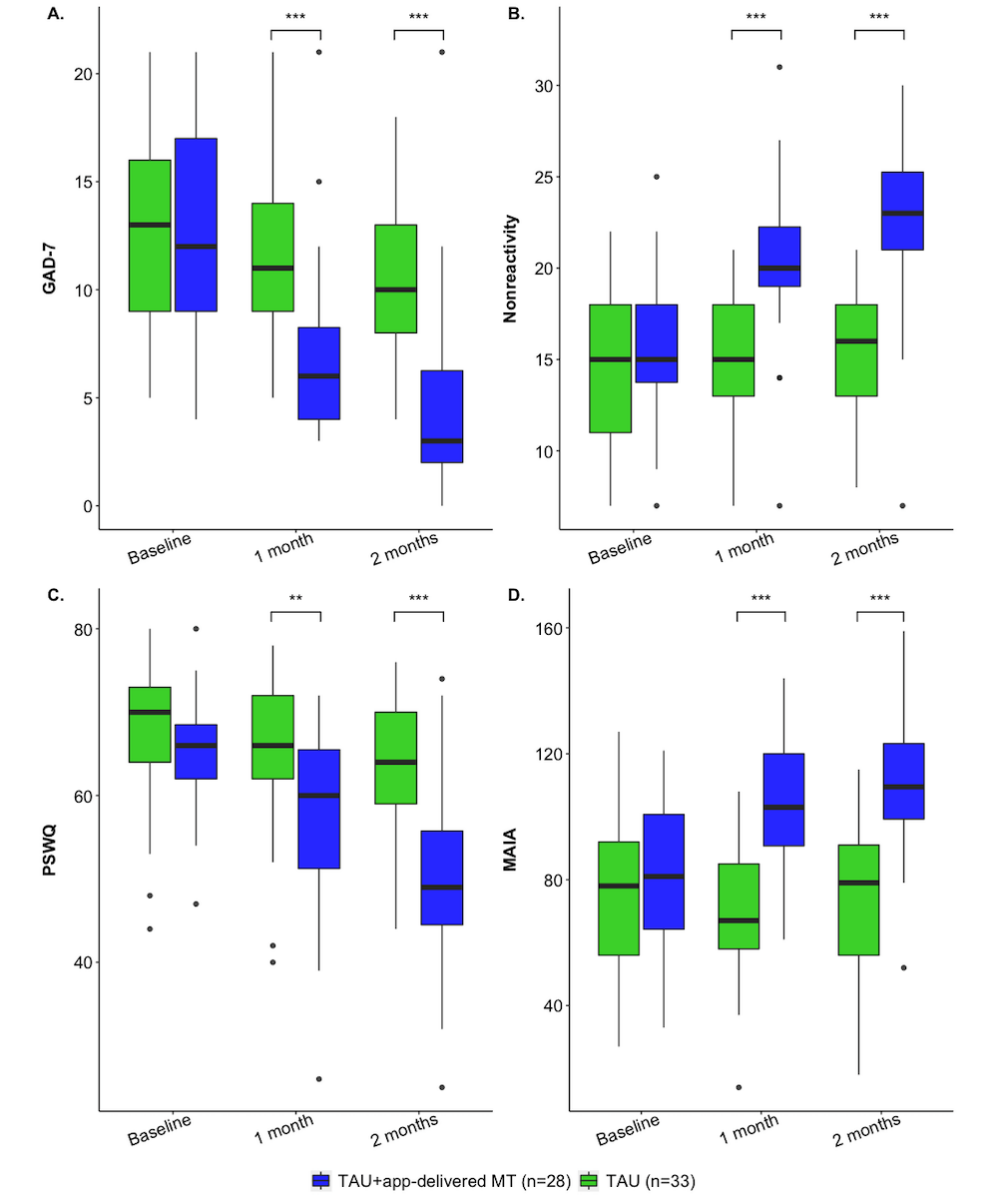
(A) Change in GAD-7 scores. (B) Change in nonreactivity scores. (C) Change in PSWQ scores. (D) Change in MAIA scores. GAD-7: Generalized Anxiety Disorder 7-item; MAIA: Multidimensional Assessment of Interoceptive Awareness; PSWQ: Penn State Worry Questionnaire.

Baseline GAD-7 scores indicated moderate (36/63, 57%) to severe (24/63, 38%) anxiety in individuals with GAD (TAU+app-delivered MT: median 12, IQR 8; TAU: median 13, IQR 7). To examine the effect of MT on reduction in anxiety, we fitted a robust mixed ANOVA with group as the between-subjects factor, time as the within-subjects factor, and GAD-7 score as the dependent variable. We found a main effect of group (*F*_1,39.99_=22.54; *P*<.001) and time (*F*_2,33.49_=29.98; *P*<.001), with a significant group×time interaction (*F*_2,33.49_=11.19; *P*<.001). At 1 month after treatment initiation, there was a significant difference between groups (*P*<.001; *r*=0.59) and the Hodges-Lehmann estimate, the nonparametric estimate of population change, was 5 (95% CI 4-7). The TAU+app-delivered MT group reported a median reduction in anxiety scores of 5 (IQR 7.3; *P*<.001; *r*=0.89) compared with no change in the TAU group. At 1 month, the calculated RCI for the TAU+app-delivered MT group was –4.6 and a reliable change was seen in 64% (18/28) of the participants, while the calculated RCI for TAU was 0. A significant between-group difference (*P*<.001; *r*=0.68) was maintained at 2 months, and the Hodges-Lehmann estimate was 6 (95% CI 5-8; Figure 3); the TAU+app-delivered MT group reported a median reduction in anxiety scores of 8.5 (IQR 6.5; *P*<.001; *r*=0.96), while the TAU group reported a median reduction of 1 (IQR 5; *P*=.01; *r*=0.37), representing a 67% versus a 14% reduction. The RCI was –7.9 for the TAU+app-delivered MT group and reliable change was seen in 54% (15/28) of the participants. The RCI was –0.9 for the TAU group. See Table 2 for medians and IQRs, in addition to the means and SDs.

**Table 2:** Group-wise results for General Anxiety Disorder 7-item, nonreactivity subscale, Penn State Worry Questionnaire, and Multidimensional Assessment of Interoceptive Awareness (N=61).

Timepoints		TAU^a^+app-delivered MT^b^ participants (n=28)			TAU participants (n=33)			*P* value^c^		Effect sizes (*r*)
		Values, median (IQR)	Values, mean (SD)	Values, median (IQR)		Values, mean (SD)				
**Generalized Anxiety Disorder-7**										
	Baseline	12.0 (8.0)	12.9 (4.8)	13.0 (7.0)		12.6 (4.3)	>.99		0	
	1 month	6.0 (4.3)	7.0 (4.1)	11.0 (5.0)		12.0 (3.7)	<.001		0.59	
	2 months	3.0 (4.3)	4.8 (4.1)	10.0 (5.0)		10.6 (3.5)	<.001		0.68	
	Δ^d^ at 1 month (%)	–5.0 (–49)	–5.9 (–41)	0.0 (0)		–0.6 (3)	<.001		0.53	
	Δ at 2 months (%)	–8.5 (–67)	–8.1 (–60)	–1.0 (–14)		–2.0 (–10)	<.001		0.55	
**Nonreactivity**										
	Baseline	15.0 (4.3)	15.4 (4.3)	15.0 (7.0)		14.5 (4.5)	>.99		0	
	1 month	20.0 (3.3)	20.0 (4.3)	15.0 (5.0)		15.0 (4.0)	<.001		0.53	
	2 months	23.0 (4.3)	22.5 (4.8)	16.0 (5.0)		15.6 (3.4)	<.001		0.67	
	Δ at 1 month (%)	5.0 (36)	4.6 (35)	0.0 (0)		0.4 (7)	<.001		0.48	
	Δ at 2 months (%)	7.5 (51)	7.1 (52)	1.0 (8)		1.1 (15)	<.001		0.57	
**Penn State Worry Questionnaire**										
	Baseline	66.0 (6.5)	65.4 (7.0)	70.0 (9.0)		67.8 (8.0)	.26		0.14	
	1 month	60.0 (14.3)	57.8 (11.2)	66.0 (10.0)		65.5 (8.7)	<.001		0.33	
	2 months	49.0 (11.3)	49.9 (11.5)	64.0 (11.0)		63.8 (7.9)	<.001		0.55	
	Δ at 1 month (%)	–7.5 (–11)	–7.6 (–12)	–3.0 (–4)		–2.3 (–3)	.02		0.34	
	Δ at 2 months (%)	–15.0 (–23)	–15.5 (–23)	–3.0 (–5)		–4.0 (–6)	<.001		0.56	
**Multidimensional Assessment of Interoceptive Awareness**										
	Baseline	81.0 (36.5)	80.9 (23.2)	78.0 (36.0)		75.4 (26.0)	>.99		0	
	1 month	103.0 (29.3)	103.1 (21.9)	67.0 (27.0)		69.6 (21.2)	<.001		0.67	
	2 months	109.5 (24.0)	112.2 (22.8)	79.0 (35.0)		74.3 (23.4)	<.001		0.87	
	Δ at 1 month (%)	22.0 (25)	22.2 (39)	0.0 (0)		–5.8 (–4)	<.001		0.60	
	Δ at 2 months (%)	26.0 (29)	31.3 (53)	–2.0 (–2)		–1.1 (1)	<.001		0.85	

^a^TAU: treatment as usual.

^b^MT: mindfulness training.

^c^Adjusted *P* values represent between-group comparisons.

^d^Δ: change between baseline and posttreatment.

#### Effects of Intervention on Nonreactivity (FFMQ Subscale)

To examine changes in nonreactivity, we performed a robust mixed ANOVA with group as the between-subjects factor, time as the within-subjects factor, and nonreactivity score as the dependent variable. This demonstrated a main effect of group (*F*_1,39.38_=34.06; *P*<.001) and time (*F*_2,28.75_=24.77; *P*<.001), with a significant group×time interaction (*F*_2,28.75_=23.23; *P*<.001). At 1 month after treatment initiation, there was a significant difference between the groups (*P*<.001; *r*=0.53) and the Hodges-Lehmann estimate was –5 (95% CI –7 to –3). The TAU+app-delivered MT group reported a median increase of 5 (IQR 6.3) in nonreactivity scores (*P*<.001; *r*=0.95), whereas participants in the TAU group reported no change (Figure 3). A significant between-group difference (*P*<.001; *r*=0.67) was maintained at 2 months, and the Hodges-Lehmann estimate was –7 (95% CI –9 to –5); the TAU+app-delivered MT group reported a median increase of 7.5 (IQR 6) in nonreactivity scores (*P*<.001; *r*=0.95), while a median increase of 1 (IQR 6; *P*=.43, *r*=0.14) was seen in the TAU group.

#### Effects of Intervention on Worry (PSWQ)

To examine the effects of MT on worry, we ran a robust mixed ANOVA with group as the between-subjects factor, time as the within-subjects factor, and PSWQ score as the dependent variable. This revealed a main effect of group (*F*_1,37.85_=19.66; *P*<.001) and time (*F*_2,27.12_=34.78; *P*<.001), with a significant group×time interaction (*F*_2,27.12_=10.30; *P*<.001). Participants in the TAU+app-delivered MT group reported a median reduction in worry scores of 7.5 (IQR 8.5) at 1 month (*P*<.001; *r*=0.67; Figure 3), whereas the TAU group reported a median reduction of 3 (IQR 4; *P*=.01; *r*=0.44). There was a significant between-group difference (*P*<.001; *r*=0.55) at 2 months after treatment initiation, and the Hodges-Lehmann estimate was 14 (95% CI 9 to 19); the TAU+app-delivered MT group reported a median reduction in worry scores of 15 (IQR 14.3; *P*<.001; *r*=0.88) compared with a median reduction of 3 (IQR 6) reported by the TAU group (*P*<.001; *r*=0.61).

#### Effects of Intervention on Interoceptive Awareness (MAIA)

To examine changes in interoceptive awareness, we fitted a robust mixed ANOVA with group as the between-subjects factor, time as the within-subjects factor, and MAIA score as the dependent variable. We found a main effect of group (*F*_1,39.19_=22.53; *P*<.001) and time (*F*_2,29.93_=10.79; *P*<.001), with a significant group×time interaction (*F_2_*_,29.93_=12.45; *P*<.001). At 1 month after treatment initiation, participants in the TAU+app-delivered MT group reported a median increase of 22 (IQR 30) in interoceptive awareness scores (*P*<.001; *r*=0.72), whereas the TAU group reported no change (median 0, IQR 18) in interoceptive awareness scores (*P*=.71; *r=*0.07; Figure 3). At 2 months, there was a significant between-group difference (*P*<.001; *r*=0.87), and the Hodges-Lehmann estimate was –13 (95% CI –15 to –10); the TAU+app-delivered MT group reported a median increase of 26 (IQR 28.5) in interoceptive awareness scores (*P*<.001; *r*=0.85), whereas the TAU group reported a median reduction of 2 (IQR 12; *P*>.99; *r*=0).

#### Mediation Analysis

**Figure 4:**
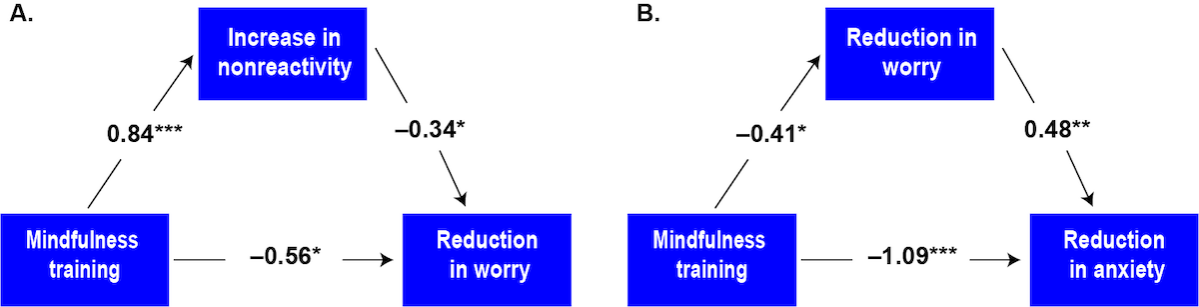
(A) Longitudinal causal mediation model with standardized regression coefficients illustrates how an increase in nonreactivity mediates the effects of mindfulness training on reduction in worry. (B) Longitudinal causal mediation model with standardized regression coefficients illustrates how a reduction in worry mediates the effects of mindfulness training on reduction in anxiety. *P<.05; **P<.01; ***P<.001.

Model A (Figure 4) shows the direct effect of MT on the reduction in worry and its indirect effect through nonreactivity. Reduction in worry and an increase in nonreactivity were defined as change in the PSWQ at 1 month and change in the nonreactivity scale from the FFMQ at 2 months after treatment initiation. Mediation analysis indicated that MT was related to a significant reduction in worry at 2 months with a direct effect of β=–.56 (SE=0.25; *P*=.03). MT also significantly increased nonreactivity (β=.84; SE=0.21; *P*<.001), which was significantly related to reduction in worry at 2 months (β=–.34; SE=0.14; *P*=.01). This implies that the relationship between MT and reduction in worry was partially mediated by an increase in nonreactivity (β_indirect effect_=.84×–.34=–.29; 95% CI –0.68 to –0.04; *P*=.02). The total effect of MT on reduction in worry at 2 months was estimated to be β=–.85 (SE=0.23; *P*<.001). No effects were observed in the control group.

Model B (Figure 4) shows the relationship between MT and reduction in anxiety with a direct effect and an indirect effect mediated by a reduction in worry. These indicate that MT also had a significant impact on reduction in anxiety at 2 months, with a total effect of β=–1.28 (SE=0.26; *P*<.001). The direct effect of MT was estimated to be β=–1.09 (SE=0.25; *P*<.001). MT was related to significant reductions in worry at 1 month (β=–.41; SE=0.20; *P*=.04), whereas the latter was significantly related to reductions in anxiety at 2 months (β=.48; SE=0.16; *P*=.004). Thus, reductions in worry partially mediated the relationship between MT and reduction in anxiety (β_indirect effect_=–.41×.48=–.19; 95% CI –0.40 to –0.02; *P*=.03). No effect was observed in the control group.

#### Engagement

To evaluate program engagement, we calculated the median and IQR. At 1 month, the median number of modules completed was 18 (IQR 16.3). At 2 months, it was 25.5 (IQR 17), and 46% (13/28) participants completed the program. To explore the association between anxiety and module completion, we fitted a robust regression model and found that for each additional completed module demonstrating further progression through intervention, anxiety scores decreased by 1.37 (β=–1.37; SE=0.23; *P*<.001). The adjusted *R*^2^ value for this model was 0.25.

### Number Needed to Treat

At 2 months after treatment initiation, we found an NNT of 1.6: 64% (18/28) of the participants achieved remission in the TAU+app-delivered MT group compared with 3% (1/33) in the TAU group.

### Risk of Bias

Using the Cochrane Collaboration criteria for evaluating bias [65], we found a low risk of bias in 6 of 7 categories, including random sequence generation, allocation concealment, blinding of outcome assessment, incomplete outcome data, selective reporting, and other bias. Blinding of participants and personnel was deemed to have a medium risk of bias because the project director and participants were unblinded to group allocation.

## Discussion

### Principal Findings

Anxiety is a debilitating and difficult-to-treat condition that affects hundreds of millions of people worldwide. Using a theory-based approach (targeting reinforcement learning), we developed a digital therapeutic that demonstrated a significant and clinically meaningful reduction in anxiety in individuals with GAD (NNT=1.6) [66]. This was confirmed by our finding that anxiety decreased with further progression through the program through the completion of more modules. Furthermore, we determined a potential mechanism underlying its effect: increases in mindfulness mediated decreases in worry and decreases in worry mediated reductions in anxiety. These effects were specific to the MT intervention. The mediation effect was significantly higher at 2 months (45%) than at 1 month (15%), which is consistent with participants having more exposure to the treatment (ie, a dose effect), a sleeper effect [67-69], or a combination of the two. These results are in direct alignment with the theoretical underpinnings that anxiety can be perpetuated through negative reinforcement—worry can feed back and perpetuate anxiety by introducing the *reward* of feeling more in control or temporarily distracting an individual from the aversive feelings of anxiety [14,15,21,42,70].

### Treatment for Anxiety

Anxiety treatment has largely relied on antidepressant medications and psychotherapy (eg, CBT). These have yielded medium effect sizes for anxiety [9]. RCI is increasingly being used in treatment studies to assess whether changes are clinically significant [63]. A longitudinal study of low-intensity CBT using an RCI of ≤5 demonstrated a reliable change in 43.8% (181/439) of the participants [71]; our study found that 64% (18/28) and 54% (15/28) of the participants demonstrated reliable change at 1- and 2-months posttreatment using RCIs of 4.6% and 7.9%, respectively. For GAD, the NNT with antidepressants was 5.15. In this study, we found that specifically targeting a mechanistic pathway yielded large effect sizes with an NNT of 1.6. A previous single-arm pilot study in physicians with comparable levels of anxiety (median baseline GAD-7 score of 11.5) showed a similar magnitude of reduction in anxiety (57% reduction at 3 months) using the same app-delivered MT program [42]. This randomized controlled trial extends previous results and broadens these findings beyond anxious physicians to individuals with moderate to severe anxiety.

### The Psychological Mechanisms of Anxiety

Potential mechanisms underlying anxiety have been hypothesized for over a century; yet, refinement in recent decades has opened the door for specific hypothesis testing [14,15]. For example, Mkrtchian et al [18] recently demonstrated avoidance as a part of reinforcement learning pathways in individuals with anxiety disorders. Our findings provide an important extension of these results by showing that targeting worry and avoidance yields clinically meaningful reductions in anxiety. These results and the finding that individuals reported increases in interoceptive awareness (measured by the MAIA) are in line with broader theoretical mindfulness frameworks that suggest that MT helps individuals learn to become more aware of and observe unpleasant emotions with awareness imbued with curiosity [72].

### App-Delivered MT Targets Worry

MT may promote decentering, defined as a “metacognitive capacity to observe items that arise in the mind as mere psychological events” [73]. Decentering may help individuals disengage from perseverative worry habit loops that are perpetuated through reinforcement learning [46-48,74]. Our findings show that increases in mindfulness directly mediate the effects of app-delivered MT on reductions in worry. This may be the case possibly because of helping individuals step out of perseverative worry habit loops that are at the core of GAD and, in doing so, reduce their reinforcement. Furthermore, our results show that reductions in worry mediate the effects of MT on reductions in anxiety.

### Practical Implications

The high prevalence of anxiety “vastly exceeds the capacities of mental health services,” and this gap has only increased over the past several years [75]. App-based digital therapeutics offer a viable and practical route toward augmenting traditional mental health care and, in some cases, serve as a first-line treatment [76]. For example, if a patient in a primary care clinic screens positive for anxiety, an evidence-based digital therapeutic such as the one described in this study can be offered as an augmentation to standard medication treatment, or in some cases, it can be offered if a trial of medications has yielded suboptimal results or if a patient is not interested or willing to try a medication as an alternative. In addition, for individuals who are concerned about the confidentiality of mental health care (eg, feeling the need to ask a boss for regular time off for therapy visits), Health Insurance Portability and Accountability Act compliant digital therapeutics can offer discretion, privacy, and convenience. As integrative care models (eg, embedding psychiatric or psychological services within primary care clinics) gain momentum, one of the primary limitations is the cost and availability of trained therapists. However, because 85% of the US population has a smartphone, digital therapeutics may be able to serve as the *mobile* component of an integrative care clinic at a low cost, filling in for the lack of physical space and trained mental health clinicians [76]. In addition, in corporate settings where employers are increasingly aiming to meet the mental health needs of employees, it may be possible to quickly and confidentially deploy evidence-based digital therapeutics to help employees with mild or moderate anxiety. For employees with severe anxiety, who may have to wait several months to see their doctor or to obtain a mental health referral, a digital therapeutic may serve as a bridge to therapy or even a first-line treatment.

### TAU Condition

We chose the GAD-7 as an outcome measure because it is widely used in clinical practice, yielding results that are interpretable in nonresearch settings. We chose TAU because clinicians deliver standard treatment, such as prescribing a medication, and bolster these with an additional medication or recommendation for psychotherapy if a patient does not achieve a reduction in symptoms (ie, the TAU+X *add on* model). Although far from perfect as a control condition, TAU is standardly used in pragmatic clinical trials for these and other reasons [77,78].

Although the TAU group showed a significant decrease in anxiety symptoms (14%), there may be several reasons why TAU failed to show a greater reduction in symptomatology or achieve remission (3% vs 64%). These include a higher NNT for current medications and current models in which medical practices are designed more as a *sick care* model, in which acute, physically based issues are prioritized over mental health despite clear advantages of integrating mental health care into primary care settings [79,80]. This study demonstrated a clear proof-of-concept trial of a mindfulness-based digital therapeutic to deliver specific theory-driven and mechanistically based treatment in a clinically relevant setting. Furthermore, we aimed to recruit a real-world population by minimizing the exclusion criteria, such as comorbid disorders. In this study, most of the individuals (84%) presented with comorbid disorders, such as depression, which is consistent with how individuals present in primary care settings and to treatment specialists.

### Limitations

This study has several strengths and notable findings, including designing for real-world applicability (eg, including individuals with comorbid disorders and concomitant medication treatment), accounting for engagement, assuring adequate sample sizes, registering outcomes, and minimizing the risk of bias. However, this study has some limitations. The TAU+ model was chosen to closely match the treatment a patient would encounter in a clinical setting. Whereas standard clinical care is highly variable, the study was designed such that randomization would ensure that this variability was equally distributed between the groups. Future studies using an active comparator (eg, CBT-based app) to control for attentional effects, longer follow-up periods, and incorporating multiple sites are needed to confirm the efficacy of this program. Second, this study sample comprised 90% (57/63) women. Although future sex-balanced studies are needed to determine the generalizability of these findings, women are twice as likely to develop an anxiety disorder and have a higher lifetime prevalence of GAD (7.1% vs 4.2%) than men [81,82]. Third, this study was designed to evaluate anxiety symptoms at 2 months after treatment initiation. Long-term follow-up studies are necessary to establish the long-term effects. Furthermore, although this study identifies potential psychological mechanisms of app-delivered MT, such as increased mindfulness mediating decreases in worry and anxiety, future studies are needed to explore its neurobiological mechanisms. In addition, studies performed in research laboratory settings (eg, National Institutes of Health stages I and II) may select more motivated individuals in general. Although randomization controls for between-group differences, real-world efficacy (National Institutes of Health stage III) is needed as the next step to determine efficacy in clinical settings.

### Conclusions

In summary, for a large portion of the world’s population that is affected by moderate to severe anxiety, targeted and mechanistically based treatments are needed. By combining theory and a new field of treatment delivery (digital therapeutics), we found that app-delivered MT significantly reduced anxiety, and its effects were mediated by increases in psychological nonreactivity and reductions in worry, suggesting a specific targeting of reinforcement learning.

## Appendix

Multimedia Appendix 1 Unwinding Anxiety module outline.

Multimedia Appendix 2 Overview of the app-delivered mindfulness training program, Unwinding Anxiety.

Multimedia Appendix 3 CONSORT-eHEALTH checklist (V 1.6.1).

## Abbreviations

ANOVA: analysis of variance
CBT: cognitive behavioral therapy
FFMQ: Five Facet Mindfulness Questionnaire
GAD: generalized anxiety disorder
MAIA: Multidimensional Assessment of Interoceptive Awareness
MT: mindfulness training
NNT: number needed to treat
PSWQ: Penn State Worry Questionnaire
RCI: reliable change index
TAU: treatment as usual

## References

[1] (2017). Data Table 1: Lifetime prevalence of DSM-IV/WMH-CIDI disorders by sex and Cohort. Lifetime Prevalence of DSM-IV/WMH-CIDI Disorders by Sex and Cohort.

[2] Huang Y, Zhao N (2020). Generalized anxiety disorder, depressive symptoms and sleep quality during COVID-19 outbreak in China: a web-based cross-sectional survey. Psychiatry Res.

[3] Qiu J, Shen B, Zhao M, Wang Z, Xie B, Xu Y (2020). A nationwide survey of psychological distress among Chinese people in the COVID-19 epidemic: implications and policy recommendations. Gen Psychiatr.

[4] Asmundson GJ, Taylor S (2020). How health anxiety influences responses to viral outbreaks like COVID-19: what all decision-makers, health authorities, and health care professionals need to know. J Anxiety Disord.

[5] Salari N, Hosseinian-Far A, Jalali R, Vaisi-Raygani A, Rasoulpoor S, Mohammadi M, Rasoulpoor S, Khaledi-Paveh B (2020). Prevalence of stress, anxiety, depression among the general population during the COVID-19 pandemic: a systematic review and meta-analysis. Global Health.

[6] (2011). Generalised anxiety disorder and panic disorder in adults: management CG113. National Institute for Health and Care Excellence (NICE).

[7] McHugh RK, Whitton SW, Peckham AD, Welge JA, Otto MW (2013). Patient preference for psychological vs pharmacologic treatment of psychiatric disorders: a meta-analytic review. J Clin Psychiatry.

[8] Katzman MA (2009). Current considerations in the treatment of generalized anxiety disorder. CNS Drugs.

[9] Bandelow B, Reitt M, Röver C, Michaelis S, Görlich Y, Wedekind D (2015). Efficacy of treatments for anxiety disorders: a meta-analysis. Int Clin Psychopharmacol.

[10] Hoffman EJ, Mathew SJ (2008). Anxiety disorders: a comprehensive review of pharmacotherapies. Mt Sinai J Med.

[11] Cuijpers P, Cristea IA, Karyotaki E, Reijnders M, Huibers MJH (2016). How effective are cognitive behavior therapies for major depression and anxiety disorders? A meta-analytic update of the evidence. World Psychiatry.

[12] Van Den Berg S, Shapiro DA, Bickerstaffe D, Cavanagh K (2004). Computerized cognitive-behaviour therapy for anxiety and depression: a practical solution to the shortage of trained therapists. J Psychiatr Ment Health Nurs.

[13] Boeldt D, McMahon E, McFaul M, Greenleaf W (2019). Using virtual reality exposure therapy to enhance treatment of anxiety disorders: identifying areas of clinical adoption and potential obstacles. Front Psychiatry.

[14] Borkovec TD, Roemer L (1995). Perceived functions of worry among generalized anxiety disorder subjects: distraction from more emotionally distressing topics?. J Behav Ther Exp Psychiatry.

[15] Borkovec T, Ray W, Stöber J (1998). Worry: a cognitive phenomenon intimately linked to affective, physiological, and interpersonal behavioral processes. Cognit Ther Res.

[16] Roemer L, Salters K, Raffa SD, Orsillo SM (2005). Fear and avoidance of internal experiences in GAD: preliminary tests of a conceptual model. Cogn Ther Res.

[17] Sibrava N, Borkovec T, Davey G, Wells A (2006). The cognitive avoidance theory of worry. Worry and its Psychological Disorders: Theory, Assessment and Treatment.

[18] Mkrtchian A, Aylward J, Dayan P, Roiser JP, Robinson OJ (2017). Modeling avoidance in mood and anxiety disorders using reinforcement learning. Biol Psychiatry.

[19] (2013). Diagnostic and Statistical Manual of Mental Disorders (5th Ed.).

[20] Borkovec TD, Robinson E, Pruzinsky T, DePree JA (1983). Preliminary exploration of worry: some characteristics and processes. Behav Res Ther.

[21] Hong RY (2007). Worry and rumination: differential associations with anxious and depressive symptoms and coping behavior. Behav Res Ther.

[22] Brewer JA, Roy A, Deluty A, Liu T, Hoge EA (2020). Can mindfulness mechanistically target worry to improve sleep disturbances? Theory and study protocol for app-based anxiety program. Health Psychol.

[23] Beck AT, Emery G, Greenberg RL (1985). Anxiety Disorders and Phobias: A Cognitive Perspective.

[24] Arnsten AFT (2009). Stress signalling pathways that impair prefrontal cortex structure and function. Nat Rev Neurosci.

[25] Goyal M, Singh S, Sibinga EM, Gould NF, Rowland-Seymour A, Sharma R, Berger Z, Sleicher D, Maron DD, Shihab HM, Ranasinghe PD, Linn S, Saha S, Bass EB, Haythornthwaite JA (2014). Meditation programs for psychological stress and well-being: a systematic review and meta-analysis. JAMA Intern Med.

[26] Parmentier FB, García-Toro M, García-Campayo J, Yañez AM, Andrés P, Gili M (2019). Mindfulness and symptoms of depression and anxiety in the general population: the mediating roles of worry, rumination, reappraisal and suppression. Front Psychol.

[27] Kabat-Zinn J (1990). Full Catastrophe Living: Using the Wisdom of Your Body and Mind to Face Stress, Pain, and Illness.

[28] Brewer JA, Pbert L (2015). Mindfulness as an emerging treatment for smoking and other addictions?. J Fam Med.

[29] Brewer JA, Mallik S, Babuscio TA, Nich C, Johnson HE, Deleone CM, Minnix-Cotton CA, Byrne SA, Kober H, Weinstein AJ, Carroll KM, Rounsaville BJ (2011). Mindfulness training for smoking cessation: results from a randomized controlled trial. Drug Alcohol Depend.

[30] Mason AE, Jhaveri K, Cohn M, Brewer JA (2018). Testing a mobile mindful eating intervention targeting craving-related eating: feasibility and proof of concept. J Behav Med.

[31] Janes AC, Datko M, Roy A, Barton B, Druker S, Neal C, Ohashi K, Benoit H, van Lutterveld R, Brewer JA (2019). Quitting starts in the brain: a randomized controlled trial of app-based mindfulness shows decreases in neural responses to smoking cues that predict reductions in smoking. Neuropsychopharmacology.

[32] Servaas MN, Riese H, Ormel J, Aleman A (2014). The neural correlates of worry in association with individual differences in neuroticism. Hum Brain Mapp.

[33] Brewer JA, Garrison KA, Whitfield-Gabrieli S (2013). What about the "Self" is processed in the posterior cingulate cortex?. Front Hum Neurosci.

[34] Brewer JA, Worhunsky PD, Gray JR, Tang Y, Weber J, Kober H (2011). Meditation experience is associated with differences in default mode network activity and connectivity. Proc Natl Acad Sci U S A.

[35] Brewer JA, Garrison KA (2014). The posterior cingulate cortex as a plausible mechanistic target of meditation: findings from neuroimaging. Ann N Y Acad Sci.

[36] Garrison KA, Scheinost D, Worhunsky PD, Elwafi HM, Thornhill TA, Thompson E, Saron C, Desbordes G, Kober H, Hampson M, Gray JR, Constable RT, Papademetris X, Brewer JA (2013). Real-time fMRI links subjective experience with brain activity during focused attention. Neuroimage.

[37] Garrison KA, Santoyo JF, Davis JH, Thornhill TA, Kerr CE, Brewer JA (2013). Effortless awareness: using real time neurofeedback to investigate correlates of posterior cingulate cortex activity in meditators' self-report. Front Hum Neurosci.

[38] Hölzel BK, Carmody J, Evans KC, Hoge EA, Dusek JA, Morgan L, Pitman RK, Lazar SW (2010). Stress reduction correlates with structural changes in the amygdala. Soc Cogn Affect Neurosci.

[39] Crane RS, Kuyken W, Williams JM, Hastings RP, Cooper L, Fennell MJ (2012). Competence in teaching mindfulness-based courses: concepts, development and assessment. Mindfulness (N Y).

[40] Deady M, Choi I, Calvo RA, Glozier N, Christensen H, Harvey SB (2017). eHealth interventions for the prevention of depression and anxiety in the general population: a systematic review and meta-analysis. BMC Psychiatry.

[41] Carl JR, Miller CB, Henry AL, Davis ML, Stott R, Smits JA, Emsley R, Gu J, Shin O, Otto MW, Craske MG, Saunders KE, Goodwin GM, Espie CA (2020). Efficacy of digital cognitive behavioral therapy for moderate-to-severe symptoms of generalized anxiety disorder: a randomized controlled trial. Depress Anxiety.

[42] Roy A, Druker S, Hoge EA, Brewer JA (2020). Physician anxiety and burnout: symptom correlates and a prospective pilot study of app-delivered mindfulness training. JMIR Mhealth Uhealth.

[43] Kroenke K, Spitzer RL, Williams JB, Monahan PO, Löwe B (2007). Anxiety disorders in primary care: prevalence, impairment, comorbidity, and detection. Ann Intern Med.

[44] Sheehan DV, Lecrubier Y, Sheehan KH, Amorim P, Janavs J, Weiller E, Hergueta T, Baker R, Dunbar GC (1998). The Mini-International Neuropsychiatric Interview (M.I.N.I.): the development and validation of a structured diagnostic psychiatric interview for DSM-IV and ICD-10. J Clin Psychiatry.

[45] (2021). Qualtrics XM.

[46] Brewer JA (2019). Mindfulness training for addictions: has neuroscience revealed a brain hack by which awareness subverts the addictive process?. Curr Opin Psychol.

[47] Brewer JA, Elwafi HM, Davis JH (2013). Craving to quit: psychological models and neurobiological mechanisms of mindfulness training as treatment for addictions. Psychol Addict Behav.

[48] Brewer JA, Ruf A, Beccia AL, Essien GI, Finn LM, van Lutterveld R, Mason AE (2018). Can mindfulness address maladaptive eating behaviors? Why traditional diet plans fail and how new mechanistic insights may lead to novel interventions. Front Psychol.

[49] Garrison KA, Pal P, O'Malley SS, Pittman BP, Gueorguieva R, Rojiani R, Scheinost D, Dallery J, Brewer JA (2018). Craving to quit: a randomized controlled trial of smartphone app-based mindfulness training for smoking cessation. Nicotine Tob Res.

[50] Spitzer RL, Kroenke K, Williams JB, Löwe B (2006). A brief measure for assessing generalized anxiety disorder: the GAD-7. Arch Intern Med.

[51] Toussaint A, Hüsing P, Gumz A, Wingenfeld K, Härter M, Schramm E, Löwe B (2020). Sensitivity to change and minimal clinically important difference of the 7-item Generalized Anxiety Disorder Questionnaire (GAD-7). J Affect Disord.

[52] Ruiz MA, Zamorano E, García-Campayo J, Pardo A, Freire O, Rejas J (2011). Validity of the GAD-7 scale as an outcome measure of disability in patients with generalized anxiety disorders in primary care. J Affect Disord.

[53] Baer RA, Smith GT, Hopkins J, Krietemeyer J, Toney L (2006). Using self-report assessment methods to explore facets of mindfulness. Assessment.

[54] Baer RA, Smith GT, Lykins E, Button D, Krietemeyer J, Sauer S, Walsh E, Duggan D, Williams JM (2008). Construct validity of the five facet mindfulness questionnaire in meditating and nonmeditating samples. Assessment.

[55] Meyer TJ, Miller ML, Metzger RL, Borkovec TD (1990). Development and validation of the Penn State Worry Questionnaire. Behav Res Ther.

[56] Mehling WE, Price C, Daubenmier JJ, Acree M, Bartmess E, Stewart A (2012). The Multidimensional Assessment of Interoceptive Awareness (MAIA). PLoS One.

[57] NIMH Clinical Research Toolbox.

[58] Hodges JL, Lehmann EL (1963). Estimates of location based on rank tests. Ann Math Statist.

[59] Field A, Miles J, Field Z (2012). Discovering Statistics Using R.

[60] Jose PE (2016). The merits of using longitudinal mediation. Educ Psychol.

[61] Imai K, Keele L, Tingley D, Yamamoto T, Vinod H (2010). Causal mediation analysis using R. Advances in Social Science Research Using R.

[62] Cook RJ, Sackett DL (1995). The number needed to treat: a clinically useful measure of treatment effect. Br Med J.

[63] Wise EA (2004). Methods for analyzing psychotherapy outcomes: a review of clinical significance, reliable change, and recommendations for future directions. J Pers Assess.

[64] Jacobson NS, Truax P (1991). Clinical significance: a statistical approach to defining meaningful change in psychotherapy research. J Consult Clin Psychol.

[65] Higgins JP, Altman DG, Gøtzsche PC, Jüni P, Moher D, Oxman AD, Savovic J, Schulz KF, Weeks L, Sterne JA, Cochrane Bias Methods Group, Cochrane Statistical Methods Group (2011). The Cochrane Collaboration's tool for assessing risk of bias in randomised trials. Br Med J.

[66] Skinner BF (1963). Operant behavior. Am Psychol.

[67] Carroll KM, Ball SA, Martino S, Nich C, Babuscio TA, Rounsaville BJ (2009). Enduring effects of a computer-assisted training program for cognitive behavioral therapy: a 6-month follow-up of CBT4CBT. Drug Alcohol Depend.

[68] Carroll KM, Easton CJ, Nich C, Hunkele KA, Neavins TM, Sinha R, Ford HL, Vitolo SA, Doebrick CA, Rounsaville BJ (2006). The use of contingency management and motivational/skills-building therapy to treat young adults with marijuana dependence. J Consul Clin Psychol.

[69] Epstein DH, Hawkins WE, Covi L, Umbricht A, Preston KL (2003). Cognitive-behavioral therapy plus contingency management for cocaine use: findings during treatment and across 12-month follow-up. Psychol Addict Behav.

[70] Brewer JA, Roy A (2021). Can approaching anxiety like a habit lead to novel treatments?. Am J Lifestyle Med.

[71] Ali S, Rhodes L, Moreea O, McMillan D, Gilbody S, Leach C, Lucock M, Lutz W, Delgadillo J (2017). How durable is the effect of low intensity CBT for depression and anxiety? Remission and relapse in a longitudinal cohort study. Behav Res Ther.

[72] Desrosiers A, Vine V, Klemanski DH, Nolen-Hoeksema S (2013). Mindfulness and emotion regulation in depression and anxiety: common and distinct mechanisms of action. Depress Anxiety.

[73] Fresco DM, Moore MT, van Dulmen MH, Segal ZV, Ma SH, Teasdale JD, Williams JM (2007). Initial psychometric properties of the experiences questionnaire: validation of a self-report measure of decentering. Behav Ther.

[74] Hoge EA, Bui E, Goetter E, Robinaugh DJ, Ojserkis RA, Fresco DM, Simon NM (2015). Change in decentering mediates improvement in anxiety in mindfulness-based stress reduction for generalized anxiety disorder. Cognit Ther Res.

[75] Firth J, Torous J, Carney R, Newby J, Cosco TD, Christensen H, Sarris J (2018). Digital technologies in the treatment of anxiety: recent innovations and future directions. Curr Psychiatry Rep.

[76] Torous J, Bucci S, Bell IH, Kessing LV, Faurholt-Jepsen M, Whelan P, Carvalho AF, Keshavan M, Linardon J, Firth J (2021). The growing field of digital psychiatry: current evidence and the future of apps, social media, chatbots, and virtual reality. World Psychiatry.

[77] Löfholm CA, Brännström L, Olsson M, Hansson K (2012). Treatment-as-usual in effectiveness studies: what is it and does it matter?. Int J Soc Welf.

[78] Kazdin AE (2015). Treatment as usual and routine care in research and clinical practice. Clin Psychol Rev.

[79] Unützer J, Schoenbaum M, Druss BG, Katon WJ (2006). Transforming mental health care at the interface with general medicine: report for the presidents commission. Psychiatr Serv.

[80] Thielke S, Vannoy S, Unützer J (2007). Integrating mental health and primary care. Prim Care.

[81] Catuzzi JE, Beck KD (2014). Anxiety vulnerability in women: a two-hit hypothesis. Exp Neurol.

[82] Christiansen D, Durbano F (2015). Examining sex and gender differences in anxiety disorders. A Fresh Look at Anxiety Disorders.

