# Supplementary material for: Clinical Efficacy and Psychological Mechanisms of an App-Based Digital Therapeutic for Generalized Anxiety Disorder: Randomized Controlled Trial
Source: J Med Internet Res. 2021 Dec 2;23(12):e26987. doi: 10.2196/26987 (PMC8686411; doi:10.2196/26987)
Supplement: Multimedia Appendix 1 [file jmir_v23i12e26987_app1.pdf]

Appendix 1. Unwinding Anxiety Module Outline

Day	Title	Subject
0	Introduction and Goals	Setting goals
1	What is Anxiety?	What is anxiety and how does reward-based learning work?
2	Anxiety Triggers	Noticing our triggers and breathing into anxiety
3	Body Scan	Intro to the body scan – a simple practice to help us pay attention
4	Science Part 1: Anxiety and the Brain	How negative reinforcement loops are formed and what it feels like to be caught up
5	Being Kind to Ourselves	How to be kind to ourselves, no matter what
6	Our Inner Screaming Child	How we feed our inner screaming child and how it keeps our habits going
7	**Week in Review**	
8	Three Gears: An Overview	Three gears as a way to shift out of anxiety. We begin by noticing our anxiety habit loops
9	Second Gear	Noticing our anxiety habit loops and exploring what we get from them
10	Curiosity	Curiosity as a key ingredient for riding out our anxiety habit loops
11	Science Part 2: Substitution. Fire Analogy	Fueling the fire of anxiety by stopping the worry habit loop
12	Third Gear: RAIN	Intro to RAIN – a practical way to start riding out anxiety habit loops
13	Noting Practice	How to be mindful, one moment at a time as we go through the day
14	**Week in Review**	
15	Staying on Track	We get off track easily. How this happens and what to do about it
16	Thinking vs. Knowing	How we can shift from trying to think our way out of anxiety to simply being aware of habit loops without reacting
17	(Un)resistance	Forcing ourselves to not be anxious can be exhausting. How do we roll with resistance?
18	Anxiety is Like a Hot Coal	How we hold onto bad habits, and how mindfulness can help us drop them, literally
19	The Committee in Our Head	When it feels like you have a committee arguing in your head, how to take mindfulness on the road (or the sidewalk)
20	Radio Thoughts	We often believe everything we think. In fact, our thoughts are none of our business
21	**Week in Review**	
22	Tripping on Thoughts	How thoughts trip us up
23	Science Part 3: Change is Scary	Change is stressful – how our brains resist change and recognizing when this happens
24	Taking a Break	Sometimes we just need a break. Here's how
25	Finding Our Flow	Exploring flow, the opposite of anxiety
26	Motivation	Building and keeping our momentum to change our habits for good
27	Faith	How we can look at our own experience to build the faith we need to succeed
28	**Week in Review**	
29	Deep Anxiety	This is a process. The importance of practicing patience and self-kindness
30	The Beginning of the End	The end (and just the beginning)
