# Supplementary material for: Clinical Efficacy and Psychological Mechanisms of an App-Based Digital Therapeutic for Generalized Anxiety Disorder: Randomized Controlled Trial
Source: J Med Internet Res. 2021 Dec 2;23(12):e26987. doi: 10.2196/26987 (PMC8686411; doi:10.2196/26987)

Appendix 2. Overview of app-delivered mindfulness training program, Unwinding Anxiety.

Home

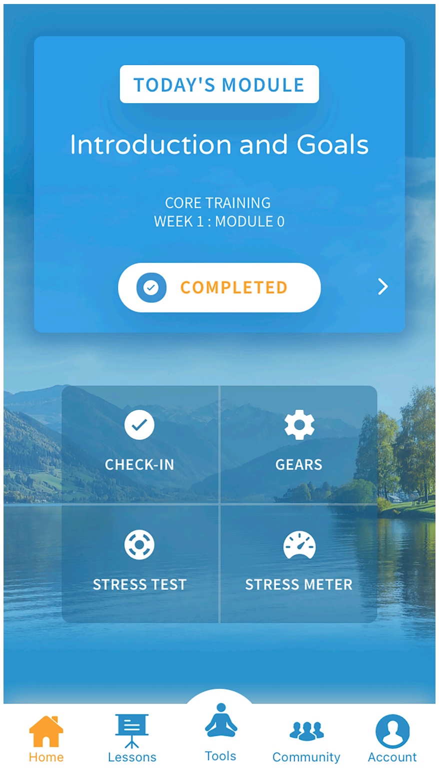

Introduction and Goals

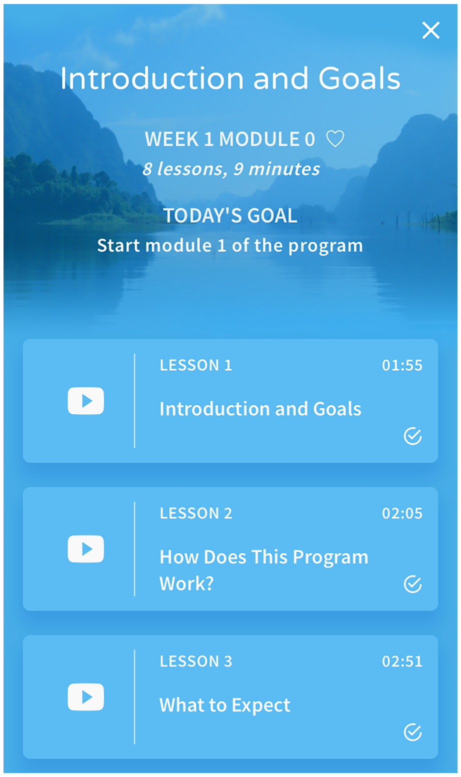

Lessons

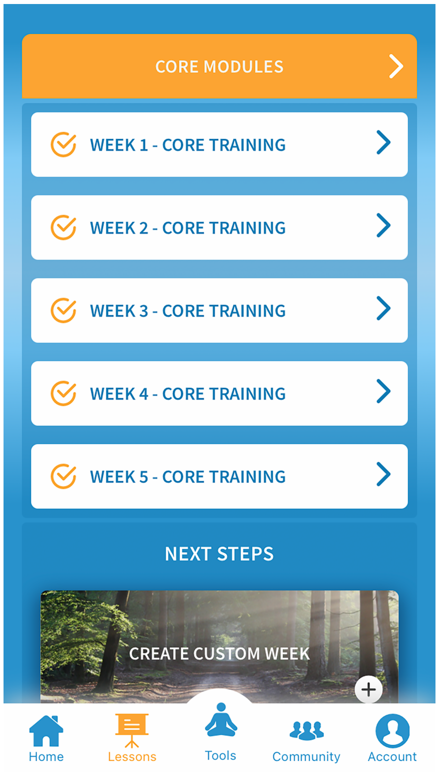

Tools

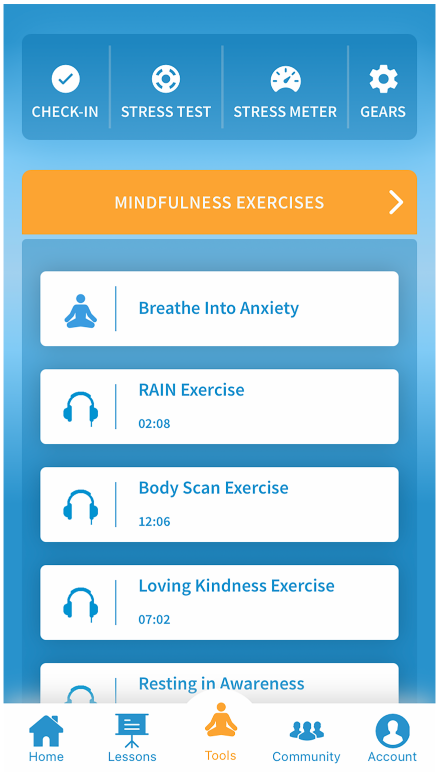

Check in

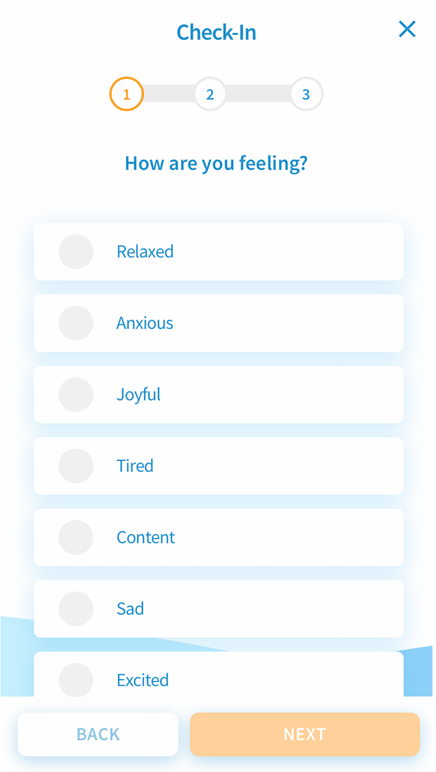

Stress Test

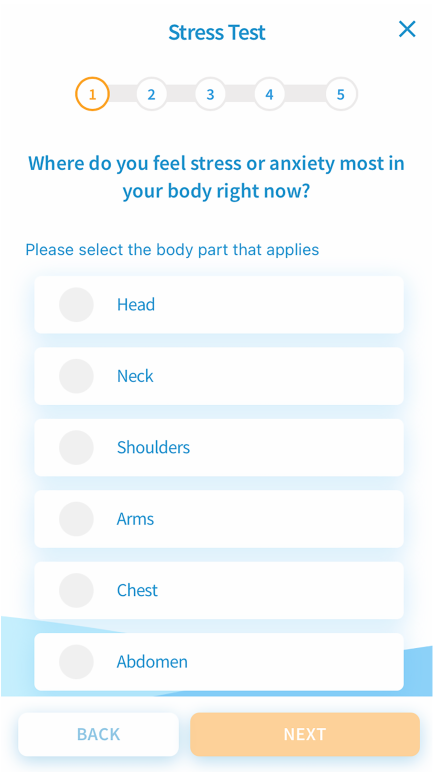

Stress Meter

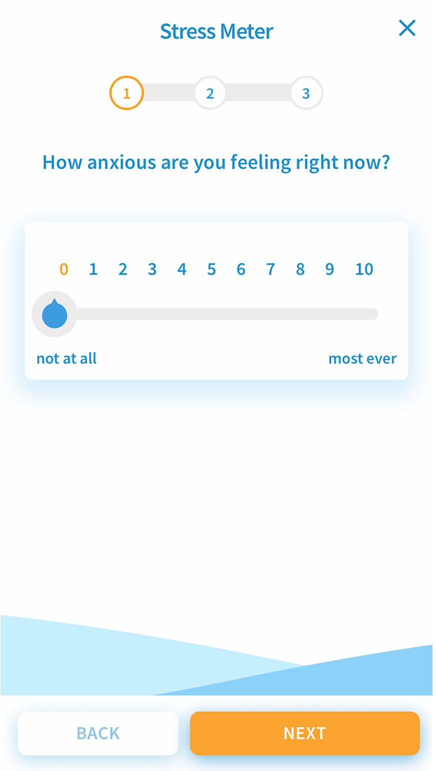

Gears

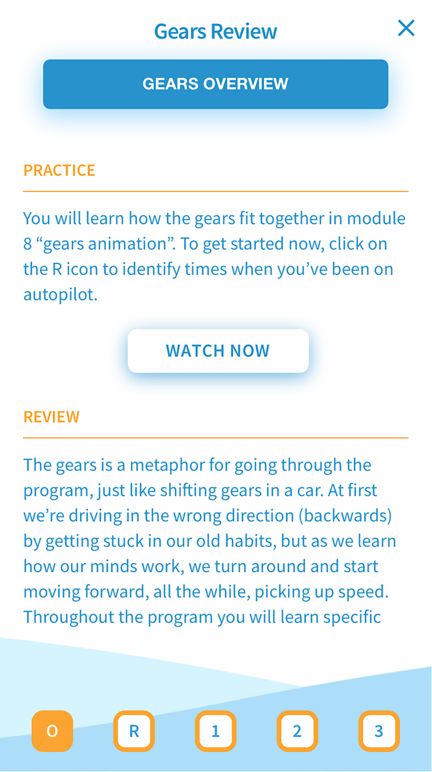

Supplement: Multimedia Appendix 2 [file jmir_v23i12e26987_app2.pdf]
